# Epithelial-Mesenchymal Transition Associates with Maintenance of Stemness in Spheroid-Derived Stem-Like Colon Cancer Cells

**DOI:** 10.1371/journal.pone.0073341

**Published:** 2013-09-09

**Authors:** Xiao-Yan Han, Bo Wei, Jia-Feng Fang, Shi Zhang, Fu-Cheng Zhang, Hai-Bo Zhang, Tian-Yun Lan, Hui-Qiong Lu, Hong-Bo Wei

**Affiliations:** 1 Central Laboratory, the Third Affiliated Hospital of Sun Yat-sen University, Guangzhou, China; 2 Department of Gastrointestinal Surgery, the Third Affiliated Hospital of Sun Yat-sen University, Guangzhou, China; University of Torino, Italy

## Abstract

Despite earlier studies demonstrating characteristics of colon cancer stem cells (CCSCs) and the role of epithelial-mesenchymal transition (EMT) in tumor development, it remains controversial as to the relationship between CCSCs and EMT. In this study, in order to present an insight into this relationship in colon cancer, we developed HCT116 and HT29 sphere models, which are known to be the cells enriching cancer stem cells. Compared to their parental counterparts, spheroid cells displayed lower homotypic/heterotypic adhesion but higher in vitro migratory/invasive capacity, as well as higher tumorigenic and metastatic potential in vivo. The spheroid cells also demonstrated down-regulated E-cadherin and up-regulated α-SMA and Vimentin expression, which is the typical phenotype of EMT. In order to explore whether this phenomenon is associated to activation of Wnt/β-catenin pathway, we detected several key signaling molecules. Compared with their parental cells, HCT116 and HT29 spheroid cells demonstrated down-regulated expression of GSK3β, but up-regulated expression of Slug and Snail. And also, the up-regulation of nucleus β-catenin in spheroid cells indicated that the free β-catenin transferred from cytoplasm to cell nucleus. Our findings indicate that spheroid cells have the characteristics of colon cancer stem cells, and EMT may account for their stemness and malignancy. And persistent activation of Wnt/β-catenin pathway may play an important role in the EMT of CCSCs.

## Introduction

Colorectal cancer is the third leading cause of cancer deaths worldwide, and the 5-year relative survival rate is only 53.8–65.2% despite diagnostic and therapeutic advances [1], [2]. Tumor recurrence and metastasis are two critical survival-influencing factors of colorectal cancer (CRC). There is a growing understanding that epithelial-mesenchymal transition (EMT) contributes to tumor invasion and metastasis [3], [4]. As we all know, EMT is a highly conserved cellular program that allows polarized, well-differentiated epithelial cells to convert to unpolarized, motile mesenchymal cells. This process is considered to promote colorectal cancer cell to invade the basement membrane and the surrounding microenvironment, such as the lymph and blood vascular systems, ultimately contribute to intra or extravasation [5].

Recently, increasing evidence suggests that tumor initiation and metastases are dependent on a small sub-population of tumor cells termed cancer stem cells (CSCs) bearing infinite self-renewal potential and the capacity to differentiate into diverse populations comprising a tumor [6], [7], [8], [9]. According to this model, cancer stem cells sustain carcinogenesis, angiogenesis, metastasis, and recurrence process of colorectal cancer [10]. In other words, cancer stem cells are in charge of the malignancy of colorectal cancer. But how do the cancer stem cells maintain their stemness, such as ability of migration, invasion, and metastasis? Many researchers observed that some cancer cells (such as breast cancer, colon cancer, etc.) can obtain the characteristics like cancer stem cells through epithelial-mesenchymal transition [11], [12], [13], [14]. That is to say, EMT may assign cancer cell the stem-like bionomics, which indicates that EMT may participate in the maintenance of stemness of CCSC. But the detailed relationship between cancer stem cells and EMT has not been reported. Meanwhile, prevalent activation of Wnt/β-catenin signaling pathway in sporadic CRC, is relevant to EMT [15]. The current study aimed to demonstrate if the bionomics of colon cancer stem cells and its maintenance of stemness are related with EMT or not, and illustrate the role of Wnt/β-catenin signaling pathway in this process.

## Materials and Methods

### Ethics Statement

All experiments involving human participants (including the collection of human colon cancer samples) have been approved by the Medical Research Ethics Committee of Sun Yat-sen University, and conducted according to the principles expressed in the Declaration of Helsinki. All participants involved in the study signed the informed consent forms. And all animal experiments were conducted according to relevant national and international guidelines. And this project was approved by the Medical Research Animal Ethics Committee of Sun Yat-sen University.

### Cell culture

HCT116 (ATCC, CCL-247) and HT29 (ATCC, HTB-38) colon cancer cell lines were maintained in DMEM/F12 supplemented with 10% FBS, 200 U/ml penicillin and 200 μg/ml streptomycin. Tumorsphere media (also called as serum free medium, SFM) was composed of DMEM/F12 media supplemented with 1×B27 (Invitrogen), EGF (20 ng/ml, Peprotech), bFGF (10 ng/ml, Peprotech), routine insulin (5 μg/ml, Invitrogen), 200 U/ml penicillin and 200 μg/ml streptomycin. For 3D floating culture, HCT116 and HT29 cells grown in two dimensional monolayer were digested with trypsin, resuspended, and then seeded at a density of 2×10^6^ cells in SFM in 100 mm ultra-low attachment dishes (Corning) at 37°C in a humidified 5% CO2/95% air atmosphere.

### Detection of CD133 expression by flow cytometry

The cells derived from monolayer cultures and suspension spheres on day 7 after primary culture were detected for the expression of CD133. The cells were washed twice in cold PBS, and subsequently cell suspensions were incubated at 4°C with 1∶10 FITC-conjugated mouse monoclonal antihuman CD133 antibody (Ab, Miltenyi biotec) for 45 minutes in the dark. After incubation, the cells were washed twice in cold PBS with 1% BSA and resuspended in 400 μl cold PBS with 1% BSA for flow cytometry analysis within 1 hour (h).

### Immunofluorescence staining

The anti-Lgr5 Ab (Abcam), anti-CK20 Ab, anti-E-cadherin Ab, anti-β-catenin Ab, anti-α-SMA Ab and anti-Vimentin Ab (Santa Cruz Biotechnologies) were prepared according to the Manufacturer's protocols. Cells were fixed with 4% of paraformaldehyde and permeabilized with 0.05% of Triton X-100 in PBS at room temperature for 20 min. Samples were blocked with 1% of bovine serum albumin (Sigma) and incubated with appropriate primary antibody at 37°C for 1 h. After washing extensively, they were incubated with Alexa Fluor-488 goat anti-mouse IgG (Invitrogen) or Fluor-Cy3 goat anti-rabbit IgG (Jackson Immunoresearch) at 37°C for 1 h. Counterstaining of nuclei with DAPI (Invitrogen) was also performed. Cells were then washed and mounted for observation under a scanning confocal microscope (LSM-710, Zeiss). Here, throughout this manuscript, data from at least three independent experiments have been analyzed to verify reproducibility of results.

### Homogeneity and heterogeneity adhesion assay

The adhesion ability of cells to ECM was tested using fibronectin (FN)-coated 96-well plates (Corning). Single-cell suspensions of spheroid and adherent cells were plated (10^5^/well), and incubated at 37°C for 2 h, then washed with PBS to remove the non-adhesive cells. Delt OD of 570 nm wavelength was determined to reflect the FN-adherent cells using Cell Counting Kit-8 (Dojindo, Japan). The adhesion ability of cells to homogeneous cells was tested using monolayer cells-paved 96-well plates. Spheroid and adherent cells were plated at 10^5^ per well, and incubated at 37°C for 60 min, 90 min, and 120 min. Then the non-adhesive cells were counted and homotypic adhesive activity was calculated using the formula: homotypic adhesion (%) = (total cell number – non-adhesive cells)/total cells×100%.

### Migration and invasion assays

Migration and invasion assays were performed in 24-well transwell chambers with 8 µm-pore polycarbonate filter inserts (Corning). A total of 5×10^4^ or 10^5^ dissociated spheroid or adherent cells were seeded on uncoated or Matrigel-coated inserts in 100 μl of serum-free medium inserts for migration or invasion assays respectively. The lower chambers were filled with 0.5 ml of 10% FBS-supplemented DMEM/F12 medium. After 24 h and/or 48 h, cells on the upper side of the filter were removed and the cells on the lower surface of the insert were fixed and stained with crystal violet. The number of stained cells was counted under a light microscope. Assays were performed in triplicates.

### Western blot analysis

GSK3β, β-catenin, Slug, and Snail expression were analyzed in total cell extracts or in nucleus extracts separated by 10% SDS-polyacrylamide gel electrophoresis and transferred to Polyvinylidene Fluoride Membrane (Millipore). The membranes were washed in Tris-buffered saline with Tween (TBST, composed of 10 mM Tris-HCl, pH 8, 150 mM NaCl, and 0.05% Tween 20), blocked 1 h at room temperature with 5% nonfat milk in TBST, then probed 1 h at room temperature with anti-E-cadherin Ab, anti-α-SMA Ab, anti-Vimentin Ab, anti-GSK3β Ab, anti-β-catenin Ab, anti-Snail Ab (Santa Cruz Biotechnologies), anti-Slug Ab (BD Pharmingen), anti-α-Tublin Ab and anti-GAPDH Ab (Peprotech). After incubation with horseradish peroxide-conjugated secondary antibody, immunoreactive proteins were detected by ECL detection system (Millipore). Quantitative analyses of immunoblotting signals were obtained via densitometry analysis using LAS4000 Image Software (Fuji Film). Protein concentrations were determined using the BCA protein assay kit (ThermoScientific Biosciences).

### In vivo tumorigenesis

To determine whether spheroid cells are more tumorigenic than their adherent counterparts in vivo, we carried out tumor development and liver metastasis experiments. Single cells were resuspended in PBS. A 100-μl suspension containing 2×10^6^ adherent cells or spheroid cells were injected subcutaneously (s.c.) into the flanks of 4- to 6-week-old male BALB/C-nu mouse, obtained from the Jackson Laboratory. Subcutaneous tumor diameters were measured with a digital caliper every two or three days, and tumor volume in mm^3^ was calculated using the formula: Volume  =  width^2^×length×0.52. Then 3 or 4 weeks later, the mice were killed and frozen tissue sections (6 μm) of subcutaneous tumors were made for pathological detection.

### In vivo liver metastasis

Liver metastatic model was used to determine the metastatic potential of spheroid cells. Liver metastases were established by intrasplenic injection of 4×10^6^ HCT116 spheroid or adherent cells and splenectomy was performed 10 min after intrasplenic injection to avoid spleen metastasis. The overall health condition was recorded in detail once a day. Liver metastasis foci were examined and measured. And tissue section was made for hematoxylin-eosin staining to confirm the pathological source.

### Statistical analysis

Statistical significance was determined by one-way ANOVA followed by Bonferroni post-hoc test for multiple comparisons or Student's t-test. For all tests, P<0.05 or <0.01 was considered statistically significant or very significant.

## Results

### Morphological and biological characterization of colonospheres

**Figure 1:**
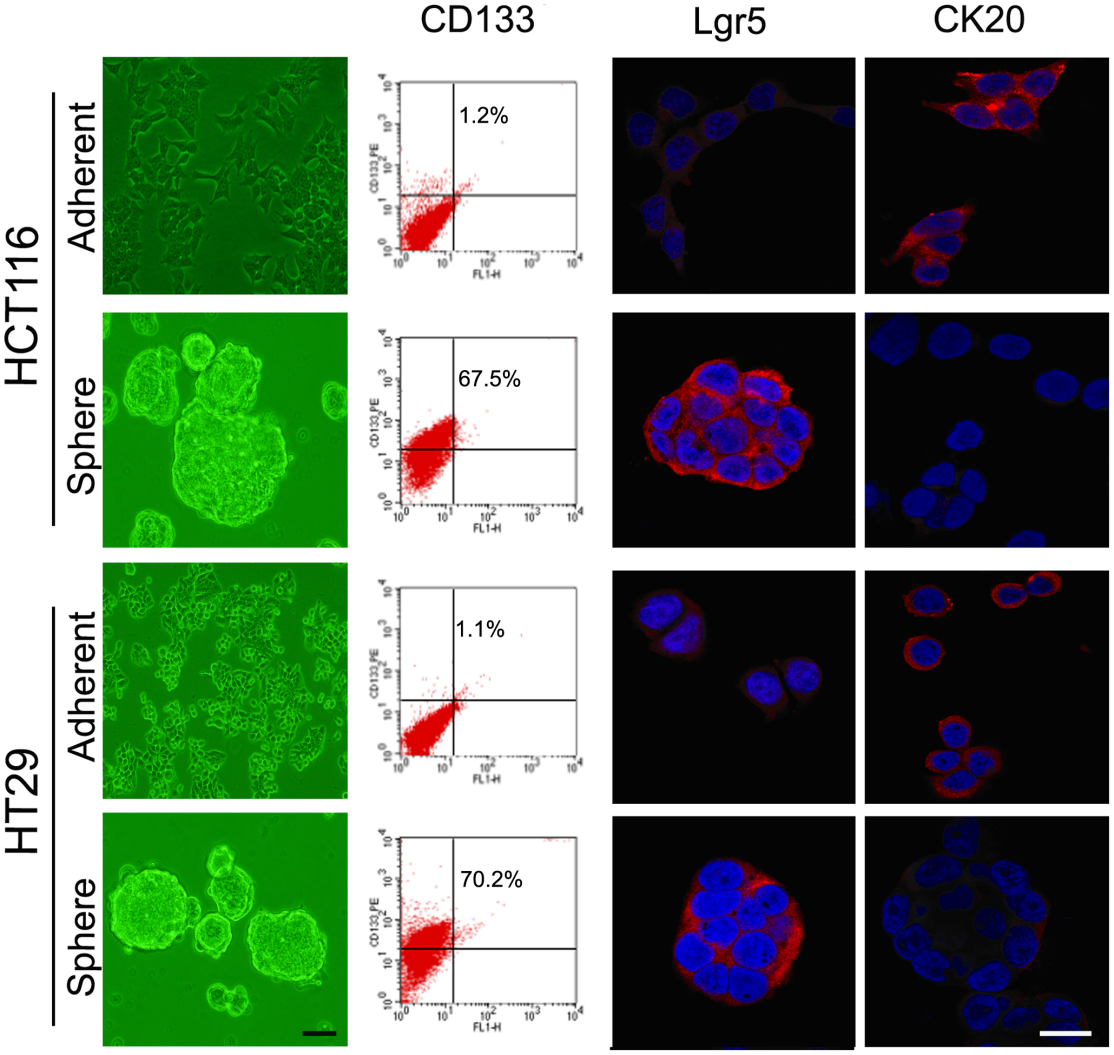
Morphological character and molecular marker expression of adherent/ spheroid cells of HCT-116 and HT29. Both HCT116 and HT29 can form large round, unattached floating colonsphere of 50–100 μm when cultured in SFM. FCM analysis showed that CD133 expression rise from ≤1.2% up to 60%–80% when cultured in SFM. And strongly expression of Lgr5 was detected by immunofluorescence staining. But as the marker of differentiated epithelial cell, CK20 expression presented adverse change.

When cultured in serum free medium supplemented with 1×B27, 20 ng/ml EGF and 10 ng/ml bFGF, both HCT116 and HT29 colon cancer cells grow in large round, unattached floating spheroid colonies (termed colonospheres) (Figure 1). Morphologically, the normal polarity of adherent cells vanished in colonspheres. Then we determined the frequency of sphere-forming cells by performing an extreme limiting dilution analysis (ELDA) for parental and spheroid-derived HCT-116 cells. The frequency to form colonsphere was found to be 5.5 fold higher in spheroid cell than parental cell line, indicating that the spheroid cell had higher self-renew capacity.

The spheroid cells showed enhanced expression of colon CSC markers-CD133 and Lgr5, but attenuated expression of differentiated epithelial cell marker CK20 compared to their corresponding parental cells. The proportion of the CD133 positive cells was determined by flow cytometry in parental and spheroid cells, they were found to be more than 80% in colonospheres, as opposed to ≤1% observed in parental cell lines (Figure 1). When the colonospheres were subjected to immunofluorescence staining for Lgr5, we observed bright Lgr5 staining on the surface of each spheroid cell indicating the presence of CSCs in colonosphere (Figure 1). The results suggest that CSCs were efficiently enriched in colonospheres. For multiple-differentiation assay, the spheroid cells were transferred serum-containing medium. Then the phenotype were detected after 4 weeks' culture. And we observed that the spheroid cells re-attached to the plastic and changed into adherent cells morphologically, along with down-regulated CD133/Lgr5 expression and up-regulated CK20 (not shown). The results suggest that spheroid cells have the capacity of multi-differentiation [10].

### Spheroid cells display lower adhesion but higher in vitro migratory/invasive capacity

**Figure 2:**
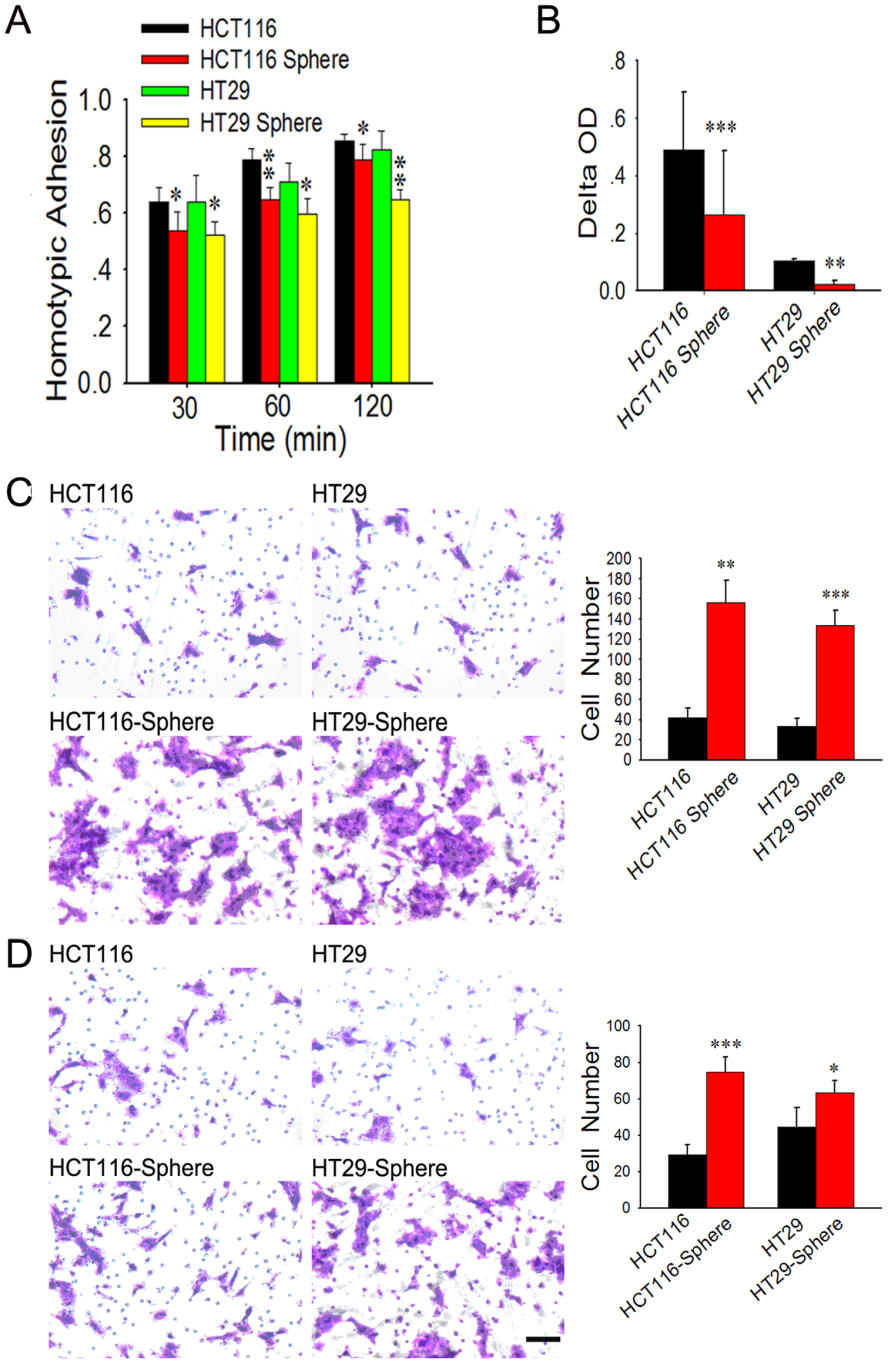
Cell adhesion and migratory/invasive capacity of adherent and spheroid cells. Spheroid cells have lower capacities of both adhesions to FN (A) and to their homogeneous neighbors (B), as compared with their parental counterparts. HCT116/HT29 spheroid cells showed a 3.8-fold (p<0.01) and 4-fold (p<0.001) increase in chemotactic potential at 24 h (C). More spheroid cells were able to invade Matrigel as compared to their parental counterparts (p<0.001 and p<0.05, respectively, D).

To corroborate malignant profile of the obtained colon cancer spheroid cells, we conducted in vitro assays to evaluate the adhesion and migratory/invasive capacity of spheroid cells in comparison with their adherent counterparts. The results of cell adhesion assay demonstrated that spheroid cells have lower capacities of both adhesion to FN (one of the key ECM components) and adhesion to their homogeneous neighbors, as compared with their parental cells (Figure 2A and 2B).

We found that spheroid cells display a significant increase in cell motility using transwell migration chambers. HCT116/HT29 spheroid cells showed a 3.8-fold (p<0.01) and 4-fold (p<0.001) increase in chemotactic potential at 24 h respectively, compared with their adherent counterparts (Figure 2C). And also, significantly more HCT116/HT29 spheroid cells were able to invade Matrigel-coated inserts in Transwell migration chambers than their parental counterparts (p<0.001 and p<0.05, respectively) (Figure 2D). These results indicate that colon cancer spheroid cells, compared to their adherent counterparts are endowed with lower adhesion, and higher migratory/invasive capacity, a functional phenotype associated with tumor aggressiveness.

### Spheroid cells possess higher tumorigenic and metastatic potential in vivo

**Figure 3:**
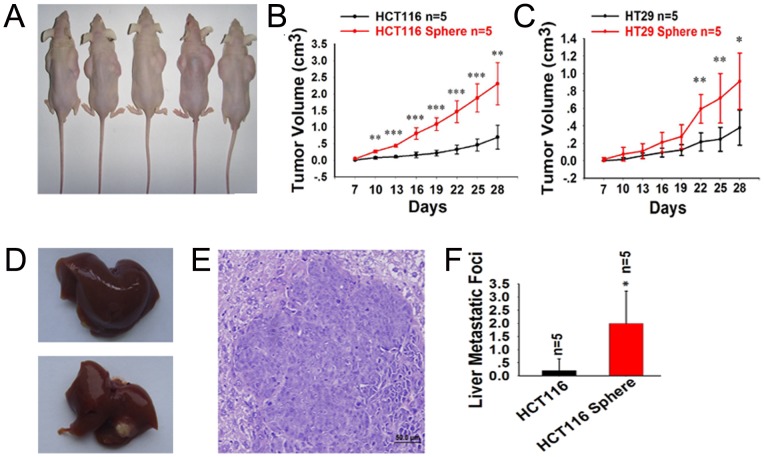
Spheroid cells possess higher tumorigenic and metastatic potential in vivo. The volumes of subcutaneous tumor in HCT116 spheroid cells group were significantly higher than that in HCT116 cells group (A, B). HT29 spheroid cells showed the similar results (C). The HCT116 spheroid cells developed more liver metastatic foci than HCT116 (D, F). Metastatic tumors were confirmed as colon cancers with hematoxylin-eosin staining (E).

To determine whether spheroid cells are more tumorigenic and metastatic than their adherent counterparts in vivo, we carried out tumor development and liver metastasis experiments. When 2×10^6^ cells were inoculated subcutaneously into nude mice, all mice developed tumors. Transplanted tumors were confirmed as colon cancers with hematoxylin-eosin staining. And the volumes of subcutaneous tumors in spheroid cells groups were significantly higher than that of adherent cells groups (Figure 3A, 3B and 3C).

As the liver metastasis was concerned, four mice (4/5) developed liver metastasis in HCT116 spheroid cells group, but only one mouse (1/5) did in adherent group (Figure 3D). Metastatic tumors were also confirmed as colon cancers with hematoxylin-eosin staining (Figure 3E). The result of liver metastatic foci counting shows the similar results (Figure 3F). The overall conditions of the mice in spheroid cells group are worse than those in adherent cells group, and some of them developed ascites and severe emaciation. The results of in vivo experiments illustrated that the spheroid-derived cells possess higher tumorigenic and metastatic capacities than their parental counterparts.

### EMT-phenotype of spheroid cells associated with Wnt/β-catenin signaling pathway

**Figure 4:**
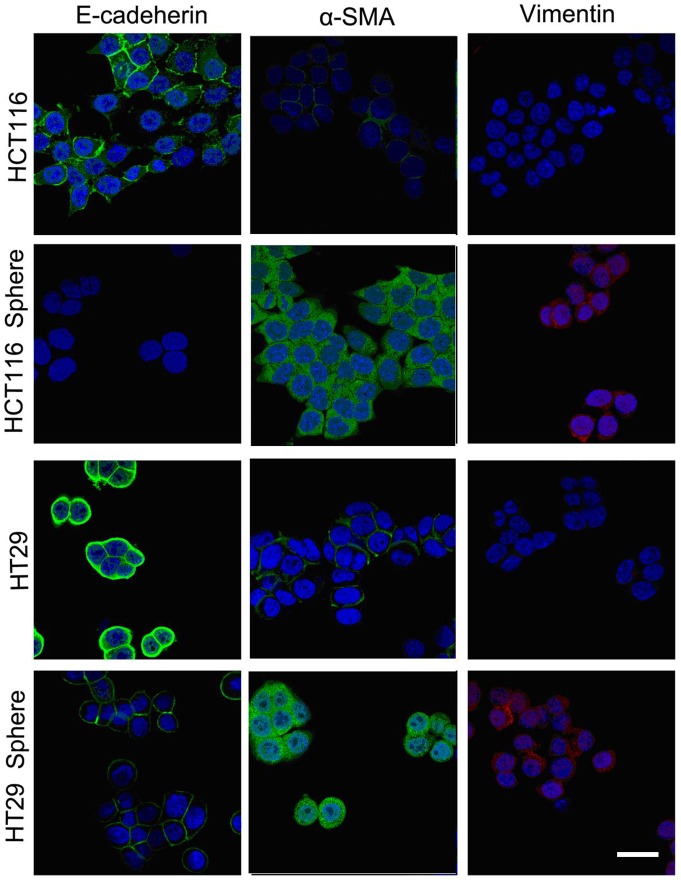
EMT-phenotype detection of spheroid/adherent cells. Representative photomicrographs depicting the higher expression of E-cadherin and the membrane localization of β-catenin in HCT116 and HT-29 adherent cells than the corresponding spheroid cells by immunofluorescence staining. And higher expression of Vimentin in spheroid cells was detected comparing to adherent cells.

**Figure 5:**
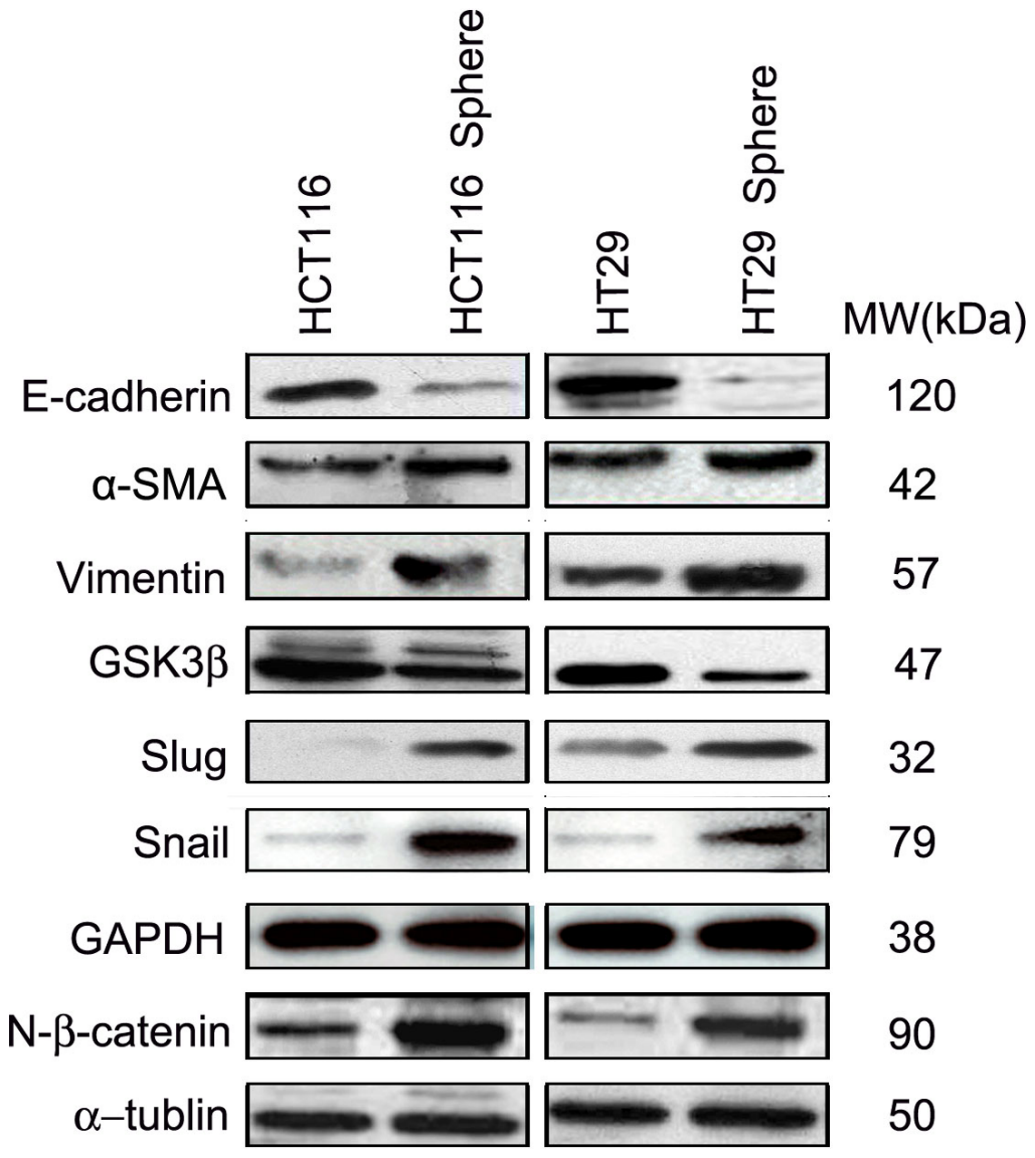
The comparative expression of different members of Wnt/â-catenin signaling pathway in parental cells and spheroid cells. The Wnt/β-catenin signaling is constitutively activated in spheroid cells, including up-regulated expression of Vimentin, Slug, Snail, and nuclear β-catenin, but down-regulated expression of E-cadherin and GSK3β, comparing with the corresponding parental cells.

To demonstrate whether there were connections between the malignant profiles of spheroid cells and EMT, we detected their EMT-phenotype. The results of IF and Western Blotting showed that down-regulation of E-cadherin expression and up-regulation of α-SMA and Vimentin expression in HCT116 and HT29 colonospheres, compared with their parental cells (Figure 4 and 5). That is to say, the spheroid cells have the characteristic of mesenchymal cells. The activation of Wnt/β-catenin pathway is reported to induce EMT [16], [17]. Then the key molecules in Wnt/β-catenin pathway were detected using Western Blotting. Compared with their parental cells, HCT116 and HT29 spheroid cells demonstrated down-regulation of GSK3β expression but up-regulation of Slug and Snail expression. And also, the up-regulation of nucleus β-catenin expression in spheroid cells indicated that the free β-catenin transfer from cytoplasm to cell nucleus (Figure 5).

## Discussion

The concept of tumor heterogeneity, which presumed there were different phenotypes in the tumor tissue, was first proposed by Fidler IJ in the 70 s of the last century [18]. The cancer stem cell theory was shaped based on this concept. Cancer stem cells have been defined as “cells within a tumor that possess the capacity for self-renewal and that can cause the heterogeneous lineages of cancer cells that constitute the tumor” at a recent American Association of Cancer Research (AACR) workshop [19]. Up to now, the existence of cancer stem cells was confirmed in several solid tumors. And the recent identification of colon cancer tumor-initiating cells adds further support to the cancer stem cell hypothesis [7]. According to this theory, the small subpopulation of cancer stem cell bears the whole characteristic of malignancy, including invasion and metastasis.

There were several methods to obtain cancer stem cells. The extensively used method to isolate or enrich colon cancer stem cells is cell sorting based on cell surface markers, such as CD133, CD44, Musasai-1, etc [20]. Then, the dye-effluxing side population cells expressing ABCG2, an ATP-binding cassette half-transporter, were isolated as cancer stem cells [21], [22], [23]. In the current study, we obtained tumor spheres from HCT116 and HT29 colon cancer cell lines by culturing in serum-free medium [24], [25]. And the following analysis indicated that CD133 and Lgr5 expression of these spheroid cells was higher than their parental counterparts, while CK20 expression was lower which represents the differentiated endothelial cell. And when were cultured in serum containing medium, the spheroid cells re-attached to the plastic and displayed down-regulated CD133/Lgr5 and up-regulated CK20 expression (not shown). The results suggest that spheroid cells have the capacity of multi-differentiation. Moreover, the spheroid cells can form the same spheres, indicating that they have the self-renew capacity. From these results, we can draw the conclusion that cancer stem cells may be enriched in these spheroid cells.

We performed a series of functional experiments, in order to take a further insight into the biological behavior of these spheroid cells. Firstly, the results of adhesion assay showed that spheroid cells demonstrate lower capacities of both adhesions to FN (one of the key ECM components) and adhesion to their homogeneous neighbors. That was to say, this kind of cells are easier to detach from the ECM and to release from the bulk tumor. And also, spheroid cells display higher capacity of in vitro migration and invasion, as compared to their adherent counterparts. As we all know, capacity of migration and solubilization of basement membrane are mandatory for the metastasis of cancer cells. So spheroid cells can migrate to the chemotactic factor more easily. And they may have more facility of enzymes secretion to solubilize the basement membrane, and easier to invade the microvessel then migrate to other organs along with blood flow. To determine whether spheroid cells are more tumorigenic and metastatic in vivo, tumor development and liver metastasis experiments were carried out. And the results showed that the volumes of subcutaneous tumor in spheroid cells groups were significantly higher than that of adherent cells. And also, liver metastatic foci counting showed the similar results. The findings of in vivo experiments illustrated that the spheroid-derived cells possess higher tumorigenic and metastatic capacities than their parental counterparts.

The cells at the invasive fronts exhibit alterations in epithelial structure and function, leading to a dissolution of adherens junctions, loss of apical-basal polarity, loss of epithelial markers, reorganization of the actin cytoskeleton, induction of a mesenchymal gene-expression program and then enhanced motility and invasion: a process referred to as epithelial-mesenchymal transition (EMT) [26], [27], [28]. Generally, E-cadherin, β-catenin and α-catenin bind to form a complex. And the connection of E-cadherin-β-catenin-α-catenin complex with Actin cytoskeleton is very important for the maintenance of cell polarity and cell adhesion. So, the proper localization of E-cadherin to adherens junctions is very important for the establishment and stabilization of these intercellular junctions amongst HCT116/HT29 cells. This is an important function, especially in the context of tumorigenesis and metastasis, because a loss of E-cadherin on the cell surface has been shown to play a role in tumor progression and metastasis [16], [29]. In our study, spheroid cells demonstrated down-regulation of E-cadherin, and up-regulation of α-SMA and Vimentin, and the latter two molecules are markers of mesenchymal cells. So, the spheroid cells display the phenotype of EMT according to our results. And the biological behaviors of spheroid cells consistent with the mesenchymal cells, such as nonpolar morphological character, lower adhesion but higher in vitro migratory/invasive capacity.

Recent studies have reported the pivotal role of Wnt/β-catenin signaling pathway in the self-renewal of epithelial stem cells [30], [31]. In contrast, dysregulation of Wnt/β-catenin signaling pathway has been implicated in colon carcinogenesis [32], [33]. It is reported that highly conserved Wnt/β-catenin signaling pathway closely associated with EMT [34]. Wnt/β-catenin signaling pathway contain many components, including Wnt proteins, Frizzled (Fz), Dishevelled (Dsh), APC/GSK3β/Axin complex, β-catenin, TCF/LEF transcription factor, and some target genes (c-myc, Cycline D, Survivin, Snail, Slug, etc.). Generally, in the absence of Wnt, the APC/GSK3β/Axin complex (destruction complex) resides in the cytoplasm, where it binds and phosphorylates β-catenin. The latter then leaves the complex to be ubiquitinated by b-TrCP (which binds to the phosphorylated ‘degron’ motif in β-catenin), is then degraded by the proteasome. In the condition of activation of Wnt/β-catenin signaling pathway, Wnt induces the association of Axin with phosphorylated LRP. The APC/GSK3β/Axin complex falls apart, and β-catenin is stabilized. The accumulated β-catenin in cytoplasm translocates to nucleus, and binds to TCF/LEF, enhances the expression of target genes, such as Slug and Snail, which induce EMT and metastasis of colon cancer [35]. The activation of Wnt signal, inhibition of APC/GSK3β/Axin complex, nuclear translocation of β-catenin, and up-regulation of Slug and Snail are key points of EMT regulation in colon cancer [36]. In our study, down-regulation of GSK3β and up-regulation of Slug and Snail expression were detected. And also, the up-regulation of nucleus β-catenin expression in spheroid cells indicates that the free β-catenin transfer from cytoplasm to cell nucleus.

In summary, our study provides evidence of the existence of cancer stem cells. The spheroid cells derived from HCT116 and HT29 cell lines enrich stem-like cells. Compared to their adherent counterparts, floating-sphere-derived cells displayed lower homotypic/heterotypic adhesion but higher in vitro migratory/invasive capacity, as well as higher tumorigenic and metastatic potential in vivo. And they demonstrate typical phenotype of EMT (down-regulated E-cadherin and up-regulated Vimentin), which may account for their malignancy. And activation of Wnt/β-catenin pathway (down-regulation of GSK3β and up-regulation of Slug, Snail and nucleus β-catenin) may play an important role in the EMT of CCSCs.
